# Neuronal nucleus and cytoplasm volume deficit in children with autism and volume increase in adolescents and adults

**DOI:** 10.1186/s40478-015-0183-5

**Published:** 2015-01-17

**Authors:** Jerzy Wegiel, Michael Flory, Izabela Kuchna, Krzysztof Nowicki, Shuang Yong Ma, Humi Imaki, Jarek Wegiel, Janusz Frackowiak, Bozena Mazur Kolecka, Teresa Wierzba-Bobrowicz, Eric London, Thomas Wisniewski, Patrick R Hof, W Ted Brown

**Affiliations:** Department of Developmental Neurobiology, NYS Institute for Basic Research in Developmental Disabilities, 1050 Forest Hill Road, Staten Island, NY 10314 USA; Department of Infant Development, NYS Institute for Basic Research in Developmental Disabilities, Staten Island, NY USA; Department of Neuropathology, Institute of Psychiatry and Neurology, Warsaw, Poland; Department of Psychology, NYS Institute for Basic Research in Developmental Disabilities, Staten Island, NY USA; Departments of Neurology, Pathology, and Psychiatry, NYU Langone Medical Center, New York, NY USA; Fishberg Department of Neuroscience, Icahan School of Medicine at Mount Sinai, New York, NY USA; Department of Human Genetics, NYS Institute for Basic Research in Developmental Disabilities, Staten Island, NY USA

**Keywords:** Autism, Neuropathology, Neuron development, Nucleus volume, Cytoplasm volume

## Abstract

**Introduction:**

Characterization of the type and topography of structural changes and their alterations throughout the lifespan of individuals with autism is essential for understanding the mechanisms contributing to the autistic phenotype. The aim of this stereological study of neurons in 16 brain structures of 14 autistic and 14 control subjects from 4 to 64 years of age was to establish the course of neuronal nuclear and cytoplasmic volume changes throughout the lifespan of individuals with autism.

**Results:**

Our data indicate that a deficit of neuronal soma volume in children with autism is associated with deficits in the volume of the neuronal nucleus and cytoplasm. The significant deficits of neuronal nuclear and cytoplasmic volumes in 13 of 16 examined subcortical structures, archicortex, cerebellum, and brainstem in 4- to 8-year-old autistic children suggest a global nature of brain developmental abnormalities, but with region-specific differences in the severity of neuronal pathology. The observed increase in nuclear volumes in 8 of 16 structures in the autistic teenagers/young adults and decrease in nuclear volumes in 14 of 16 regions in the age-matched control subjects reveal opposite trajectories throughout the lifespan. The deficit in neuronal nuclear volumes, ranging from 7% to 42% in the 16 examined regions in children with autism, and in neuronal cytoplasmic volumes from 1% to 31%, as well as the broader range of interindividual differences for the nuclear than the cytoplasmic volume deficits, suggest a partial distinction between nuclear and cytoplasmic pathology.

**Conclusions:**

The most severe deficit of both neuronal nucleus and cytoplasm volume in 4-to 8-year-old autistic children appears to be a reflection of early developmental alterations that may have a major contribution to the autistic phenotype. The broad range of functions of the affected structures implies that their developmental and age-associated abnormalities contribute not only to the diagnostic features of autism but also to the broad spectrum of clinical alterations associated with autism. Lack of clinical improvement in autistic teenagers and adults indicates that the observed increase in neuron nucleus and cytoplasm volume close to control level does not normalize brain function.

## Introduction

For 70 years brain abnormalities in autism spectrum disorder (ASD) were viewed as static [1], and age-associated alterations were beyond the scope of neuropathological studies. However, more recent clinical and morphological studies have disclosed evidence of developmental and age-associated changes and have suggested a dynamic nature of structural and functional changes throughout the lifespan of individuals with autism. The dichotomous concept of autism distinguishes between the “early-onset” pattern of clinical deficits, which emerge in the first year of life, and regressive autism, which is diagnosed in children who develop typically for the first year but lose acquired skills and develop autistic symptoms in the second year of life [2]. Development of the autistic phenotype typically takes three years and consists of several steps, suggesting a progressive involvement of brain structures and neuronal networks. The earliest signs noted by approximately 50% of parents are nonspecific and include behavioral changes ranging from marked irritability to alarming passivity, poor eye contact, and lack of response to parental voice or interaction [3]. Behavioral modifications at the age of 12 months cross several functional domains including visual attention, imitation, social responses, motor control, and reactivity [4]. Mild, atypical language deficits detected at the age of 12 months typically become severe delays by 24 months [3,5]. At three years of age, all diagnostic features, including difficulties in social reciprocity and communication, and restricted/repetitive interests, are usually present [6]. The improvement in daily living skills during adolescence and in the early 20s and the plateau in skills during the late 20s [7] indicate that the clinical phenotype is formed during early childhood but undergoes further modifications during adolescence and adulthood.

Early childhood brain overgrowth is widely cited as evidence of age-associated developmental structural alterations linked to clinical regression in autism. At birth, the head circumference of neonates later diagnosed with autism is in the range of normal, but by 1 or 2 years of age, a rapid increase is typically observed [8-10], which is followed by growth inhibition resulting in only a 2% larger brain size in autistic adults [11] or a smaller brain size in comparison to control subjects [12]. The regression with the loss of previously acquired skills, including verbal, nonverbal, and social abilities, reported in 15% to 62% of individuals with autism [13-17] suggests that clinical deterioration might be associated with structural changes. The period of acceleration of head growth overlaps with the onset of behavioral changes, whereas deceleration coincides with behavioral worsening in the second year of life [18]; however, the cellular nature of accelerated brain growth is unknown.

Among people with autism, one of the relatively common components of brain pathology is the presence of developmental abnormalities [19,20] contributing to epilepsy. However, the probability of development of epilepsy is a function of age [21], with a bimodal distribution of the onset of seizures, one peak occurring before the age of 5 years, and the other after the age of 10 [22]. The highest prevalence is observed in patients older than 12 years [23]. The frequency of epilepsy in autism has been reported to range from 5% to 38.3% [21], but long-duration video-electroencephalogram (EEG) telemetry of children with ASD and the history of regression reveal an epileptiform EEG in another 46% of ASD cases [24]. The bimodal distribution of the onset of seizures appears to be another marker of the age-associated alterations that might be triggered by biochemical and structural alterations [25].

The high prevalence of intellectual deficit and epilepsy in ASD indicates that all three disorders share elements of their pathophysiology [26], but that each of these disorders has its own timing of onset and dynamic of clinical expression. Neuropathological studies cannot monitor structural changes during the first three years when the autistic phenotype is emerging but before children have been diagnosed. However, the significantly smaller volume of neuronal soma in 14 of 16 examined brain regions in 4- to 8-year-old children with autism suggests that neuronal size alterations are present earlier than 4 years of age and contribute to the clinical phenotype [27]. The fact that only three regions show a neuronal volume deficit in 11- to 23-year-old autistic individuals indicates biochemical and structural remodeling of the brain in teenagers and young adults.

One may assume that these alterations of neuronal soma volume result from changes in the neuronal nucleus or cytoplasm, or both. Nuclear size is only partially regulated by chromatin organization [28], and experimental studies indicate that alterations of cytoplasm components have a significant impact on nuclear size and function [29]. The overall goal of this study was to establish the course of volume changes of the neuronal nucleus and soma cytoplasm throughout the lifespan, assuming that an altered trajectory reflects neuronal development and maturation defects. The broad spectrum of clinical features of autism (impaired communication and social skills, repetitive and stereotypic behaviors, intellectual deficits [30], epilepsy or epileptogenic activity in the majority of autistic individuals [25,26], and other clinical alterations) suggests multiregional rather than topographically limited brain pathology. The aim of our study of neuronal nucleus and cytoplasm volumes in 16 brain regions in autistic and control subjects was to characterize the global pattern of subcellular changes that may correspond to a broad spectrum of behavioral alterations in autism, including intellectual deficits and epilepsy. To detect differences between the course of autism-specific and control-specific nucleus and cytoplasm alterations throughout the lifespan 4- to 8-, 11- to 23-, and 29- to 60-year-old autistic and control subjects were examined.

## Materials and methods

The brain hemispheres used in this project were preserved for several parallel studies designed to detect the global pattern of developmental abnormalities of neurogenesis, migration, and dysplastic changes [19,31] and to establish the global pattern of alterations of the number and size of neurons in the cerebral cortex [27,32-35], subcortical structures, cerebellum, and brainstem [27,35]. Provisionally, 39 brains were assigned to the stereological study, including 21 brains from autistic individuals and 18 from controls. However 11 brain samples (seven autistic and four controls) were excluded because postmortem examination revealed pathology not related to autism, changes associated with mechanisms of death, or postmortem autolysis, which may distort the results of stereological estimates. Application of these criteria resulted in inclusion of brains of 14 individuals diagnosed with autism, including 10 males and four females 4 to 60 years of age, and 14 control subjects, including nine males and five females 4 to 64 years of age.

Diagnosis of autism was confirmed with the Autism Diagnostic Interview-Revised (ADI-R) [6]. The intellectual disability of eight subjects (61%) ranging from mild to severe was determined with the Wechsler Intelligence Scale for Children III and the Woodcock-Johnson Tests of Achievement-Revised. Seven of the 14 autistic subjects (50%) were diagnosed with seizures, and in five cases, death was seizure-related (36%). Neuropathological examination revealed focal developmental abnormalities of neurogenesis, migration, and dysplastic changes in the brains of 92% of autistic subjects [19].

The difference between the postmortem interval (PMI) in the control group (6–28 hours; 16.7 hours on average; standard deviation, SD ± 6.6 hours) and in the autistic group (8–50 hours; 21.9 hours on average; ± 11.4 hours) was insignificant. Also, the difference between the average weight of the brains in the autistic group (1,453 g) and in the control group (1,372 g) was insignificant. The brain hemisphere was fixed with 10% buffered formalin for an average of 408 days in the control group (range 52–1,819 days) and 905 days (range 75–4,560 days) in the autistic cohort. Neither the average time of brain hemisphere dehydration in ethyl alcohol (36 days in the control group and 38 days in the autistic group) nor the reduction of brain hemisphere weight during dehydration (47% ± 7% in the autistic and 45% ± 7% in the control group) were significantly different. Brain hemispheres were embedded in 8% celloidin [36] and cut into serial 200-μm-thick sections that were stained with cresyl violet and mounted with Acrytol.

### Stereological analysis

To establish the global pattern of neuronal volume alterations, the volume of the neuronal nucleus and cytoplasm was estimated in 16 brain structures/neuronal populations, including the amygdala, entorhinal cortex, Ammon’s horn, claustrum, caudate nucleus, putamen, globus pallidus, nucleus accumbens, thalamus including the magno- and parvocellular layers of the lateral geniculate nucleus (LGN), substantia nigra, and magnocellular basal complex (MBC); Purkinje cells and dentate nucleus in the cerebellum; and the inferior olive in the brainstem. Neurons were distinguished from glial cells by using several morphological features including neuron size, shape, and spatial orientation typical for specific layers, sectors, and brain nuclei. Additional feature were the pattern of staining and distribution of nuclear chromatin, the distinct nucleolus in the majority of examined neuronal populations, and cytoplasm morphology. Small and round nuclei with uniform, intense staining of nuclear chromatin distinguished oligodendrocytes from astrocytes, which had large round nuclei with a small amount of dispersed chromatin and an undetectable nucleolus.

The volume of neuronal soma and nucleus was determined with the nucleator method [37] using MicroBrightfield software (Stereo Investigator, version 7.003, MBF Bioscience, Williston, VT, USA). Five rays were used to estimate the volume of the cell and the nucleus. Five points of intersection of systematic randomly rotated radii with the nuclear and cell border were marked. The neuronal soma and cell nucleus volumes were estimated from the lengths of radial cellular segments. The volume of the cytoplasm was calculated as the difference between neuronal body and nucleus volume. Measurements were performed using a workstation consisting of an Axiophot II (Carl Zeiss, Goettingen, Germany) light microscope with Plan Apo objectives 1.25× (numerical aperture, N.A., 0.15) or 2.5× (N.A. 0.075) (border lines of region of interest), 40× (N.A. 0.75) or 63× (N.A. 0.9) (neuronal and nuclear measurements); a specimen stage with a three-axis, computer-controlled stepping motor system (Ludl Electronics; Hawthorne, NY, USA); and a CCD color video camera (CX9000 MBF Bioscience).

We used from four equidistant serial sections [38] in structures with relatively uniform cytoarchitecture (caudate nucleus, putamen, and globus pallidus) to 12 or 14 serial sections in more heterogeneous structures such as the amygdala or Ammon’s horn. An optical fractionator systematic random sampling scheme was applied using Stereo Investigator (MBF Bioscience). The number of virtual counting spaces was adjusted to region-specific numerical density and ranged from 59, in small neuronal groups in the MBC, to 664 for Purkinje cells with significant variations in the numerical density per counting space. To achieve a Schaeffer coefficient of error (CE) of less than 5%, the number of examined neurons per region in individual cases ranged from 153 in the MBC to 509 in the Ammon’s horn. The volume of the nucleus and cytoplasm in the cytoarchitectural subdivisions of the amygdala, entorhinal cortex, Ammon’s horn, and substantia nigra are presented as a mean value for the entire structure. Details of the applied methods and anatomical boundaries of the examined brain structures and their cytoarchitectural subdivisions have been described in detail in Wegiel et al. [27].

### Statistical analysis

This study analyzed the effect that autism has on nucleus and cytoplasm volume, with age-matched controls as a comparison group. Data have been post-stratified to adjust for the differing numbers of neurons sampled per individual so as to weight each individual equally. Any data points more than 1.5 times the interquartile range below the 25^th^ percentile or an equal amount above the 75^th^ percentile for each structure or subdivision were considered outliers and were omitted from analyses. The removed data accounted for 0.3% of all records.

Analyses were carried out separately for three groups of autistic cases: 4–8 years, 11–23 years, and 36–60 years of age. Three groups of controls were selected to provide age-matching at the group level. The age ranges for control groups were 4–8 years, 14–23 years, and 29–64 years of age. Our previous study of neuronal soma volume alterations throughout the lifespan revealed a significant neuron soma volume deficit in 14 of 16 examined regions in children with autism 4 to 8 years of age, but the number of regions with a significant deficit of neuronal soma volume decreased to three in autistic teenagers/young adults (11 to 23 years of age) and stabilized at four in subjects 36 to 60 years of age. To maintain compatibility of results from the previous study and this analysis of neuron nucleus and cytoplasm volume, the same age divisions were applied.

Comparisons were carried out across 16 regions. Adjustment for multiple comparisons was performed using the Benjamini-Hochberg method [39] to maintain a false discovery rate (FDR) of 0.05. Accordingly, p values of 0.019 or less were considered statistically significant. Analyses were conducted using versions 11.1 and 12.1 of the Stata statistical package [40,41].

Following the procedure developed in our previous analyses of the effects of potential confounders [27] including PMI (in hours), fixation time (in days), brain weight (in grams), brain weight loss during processing and dehydration (as a percentage), duration of dehydration (in days), history of seizures, and sudden and unexplained death in patients with known epilepsy (SUDEP), analyses were controlled for the log of the PMI, days of dehydration, and weight loss during dehydration.

The statistical significance of the differences in mean neuronal volumes between autistic and control brains and between pairs of age ranges was computed in general linear mixed models adjusted for the autocorrelation of measurements within each brain by using the xtmixed procedure in version 12 of the Stata statistical package [41]. This method takes into account the variance among measurements of each individual while assessing the significance of the differences between the brains of autistic and control individuals.

## Results

### Brain region–specific proportions between neuronal soma, nucleus, and cytoplasm volumes

**Figure 1:**
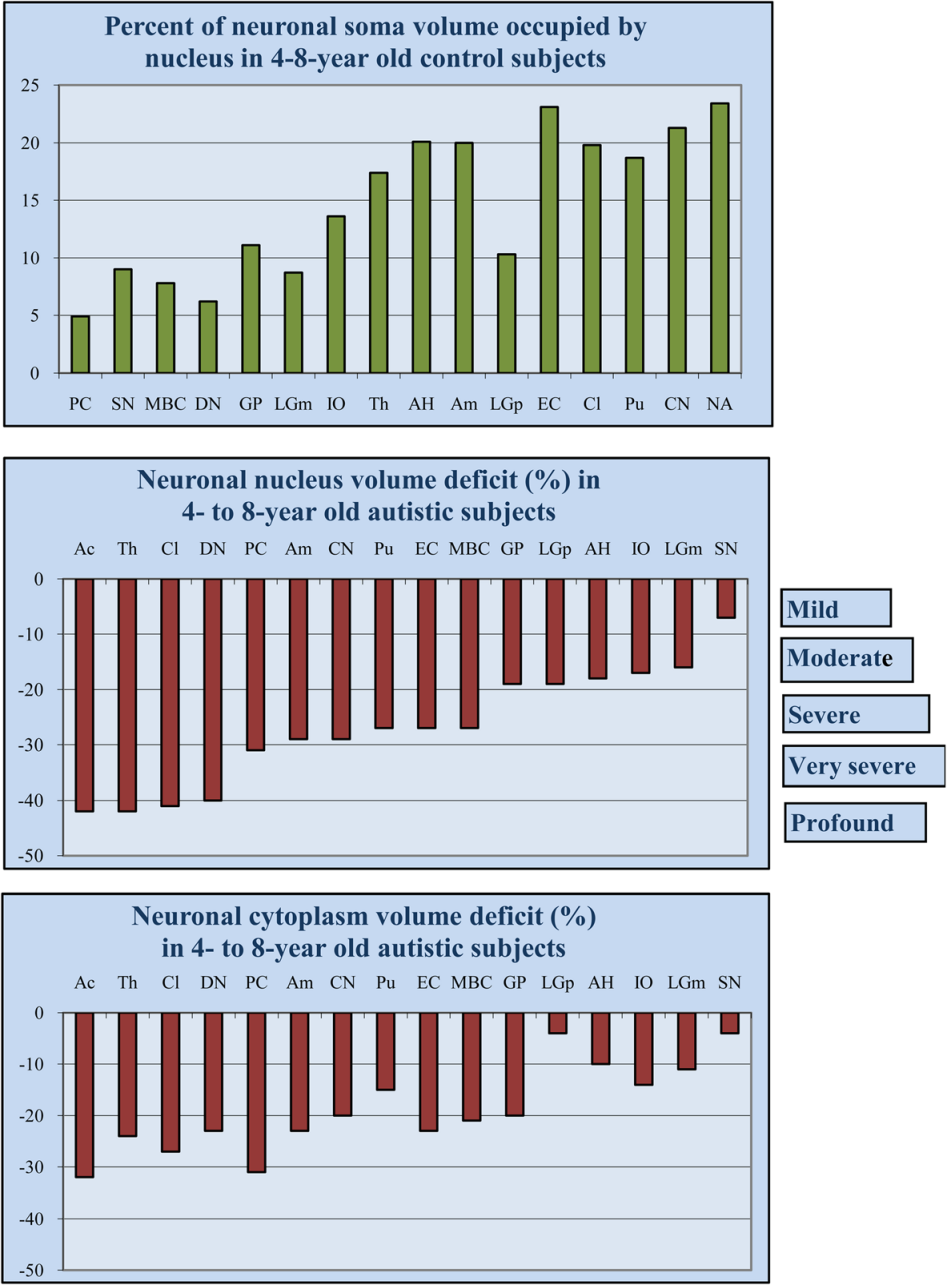
**Region-specific proportion between nucleus and neuron soma volume and the range of neuronal nucleus and cytoplasm volume deficit in children diagnosed with autism.** Percent of cell volume occupied by nucleus in 4- to 8-year-old control subjects illustrates neuron type– or region-specific proportions between cell and nucleus volume, with the smallest contribution of nucleus to neuron volume in the largest neurons (Purkinje cell, 5%), and the largest contribution in the smallest neurons (nucleus accumbens, 24%). Neuronal nucleus volume deficits (control 100%) in 4- to 8-year old autistic subjects range from mild (<10%) in the substantia nigra to profound (>40%) in the n. accumbens, thalamus, and claustrum. The cytoplasm volume deficit is less prominent and ranges from 4% in the substantia nigra to 32% in the nucleus accumbens. PC, Purkinje cell; SN, substantia nigra; MBC, magnocellular basal complex; DN, dentate nucleus; GP, globus pallidus; LGNm, magnocellular layer of the lateral geniculate nucleus; IO, inferior olive; Th, thalamus; AH, Ammon’s horn; Am, amygdala; LGNp, parvocellular layer of the lateral geniculate nucleus; EC, entorhinal cortex; Cl, Claustrum; Pu, putamen; CN, caudate nucleus; Ac, nucleus accumbens.

In 16 examined brain regions in 4- to 8-year-old control subjects, the mean volume of nuclei varied from 246 μm^3^ in small neurons in the putamen to 815 μm^3^ in the largest neurons in the substantia nigra (Table 1). The percentage of cell volume occupied by the nucleus was the smallest in large neurons, such as Purkinje cells (5%), and the largest in small neurons such as in the nucleus accumbens (24%) (Figure 1).

**Table 1:** **The difference between the mean volume of neuronal nucleus in three age groups of control and autistic subjects**

**Brain structure**	**4- to 8-year-old subjects**		**11- to 23-year-old subjects**		**29- to 64-year-old subjects**	
	**Control**	**Autism**	**Control**	**Autism**	**Control**	**Autism**
	**Mean ± SD (100%)**	**Mean ± SD** ***p*** **< (%)**	**Mean ± SD (100%)**	**Mean ± SD** ***p*** **< (%)**	**Mean (SD) (100%)**	**Mean ± SD** ***p*** **< (%)**
Nucleus accumbens	281 ± 44	163 ± 49 -42% 0.001	226 ± 52	213 ± 54 0.959 ns	239 ± 34	253 ± 7 0.224 ns
Purkinje cells	575 ± 63	394 ± 77 -31% 0.001	561 ± 83	552 ± 168 0.635 ns	569 ± 63	654 ± 189 0.368 ns
Claustrum	395 ± 32	233 ± 371 -41% 0.001	360 ± 49	255 ± 67 -29% 0.001	267 ± 16	307 ± 76 0.841 ns
Thalamus	645 ± 56	371 ± 56 -42% 0.001	439 ± 95	433 ± 77 0.186 ns	462 ± 68	581 ± 103 0.035 ns
Globus pallidus	625 ± 93	503 ± 55 -19% 0.001	417 ± 103	479 ± 95 0.611 ns	478 ± 60	489 ± 75 0.918 ns
Dentate nucleus	510 ± 168	305 ± 152 0.104 ns	448 ± 20	489 ± 122 0.312 ns	397 ± 53	501 ± 128 + 26% 0.008
Entorhinal cortex	561 ± 67	408 ± 49 -27% 0.001	502 ± 110	444 ± 98 -11% 0.002	452 ± 69	484 ± 102 0.465 ns
Amygdala	606 ± 24	431 ± 74 -29% 0.001	595 ± 60	520 ± 88 0.691 ns	548 ± 48	498 ± 116 0.184 ns
Magnocellular basal complex	656 ± 45	476 ± 45 -27% 0.001	575 ± 46	581 ± 51 0.051 ns	558 ± 116	611 ± 131 0.028 ns
Putamen	246 ± 39	180 ± 32 -27% 0.014	244 ± 52	231 ± 51 0.443 ns	213 ± 27	223 ± 34 + 5% 0.004
Caudate nucleus	256 ± 54	181 ± 52 0.269 ns	229 ± 44	234 ± 49 0.284 ns	182 ± 26	234 ± 56 + 28% 0.001
Inferior olive	613 ± 122	507 ± 125 -17% 0.001	525 ± 67	553 ± 103 0.905 ns	508 ± 68	551 ± 73 0.481 ns
Magnocellular LGN	455 ± 66	382 ± 85 0.364 ns	457 ± 33	483 ± 85 + 5% 0.001	502 ± 20	567 ± 36 + 13% 0.002
Parvocellular LGN	268 ± 50	217 ± 47 -19% 0.005	264 ± 25	263 ± 48 0.145 ns	284 ± 28	335 ± 37 + 18% 0.001
Substantia nigra	815 ± 122	756 ± 50 -7% 0.001	811 ± 114	816 ± 97 0.202 ns	646 ± 56	685 ± 65 0.057 ns
Ammon’s horn	702 ± 40	576 ± 72 -18% 0.001	702 ± 72	631 ± 79 0.246 ns	683 ± 110	706 ± 138 + 3% 0.002

Significance levels computed controlling for post-mortem interval, days of fixation, days of dehydration, and weight loss during dehydration. Mean nucleus volume ± SD; SD, standard deviation; ns, not significant; LGN, lateral geniculate nucleus.

### Nuclear and cytoplasmic volume deficits in 4- to 8-year-old autistic children

A significant, brain region–specific, developmental neuronal nucleus volume deficit was identified in 13 brain structures in autistic subjects. The neuronal nucleus volume deficit ranged from −42% in the nucleus accumbens and thalamus to −7% in the substantia nigra (Table 1; Figure 1). A profound nucleus volume deficit (>40%) was observed in the nucleus accumbens, thalamus, and claustrum; very severe (>30%) in Purkinje cells; severe (<30%) in the amygdala, putamen, entorhinal cortex, and MBC; moderate (<20%) in the globus pallidus, parvocellular lateral geniculate nucleus, Ammon’s horn, and inferior olive; and mild (<10%) in the substantia nigra. In three regions (dentate nucleus, caudate nucleus, and magnocellular layer of the LGN), the difference between the neuronal nucleus volume in autistic and in control children was high, but the significance level was >0.019 when controlling for PMI, time of fixation and dehydration, and brain weight loss during dehydration.

The mean nucleus volume deficit was significantly more prominent than the cytoplasmic volume deficit. The neuronal cytoplasm volume deficit ranged from −32% in the nucleus accumbens to −4% in the substantia nigra (Figure 1). Only in Purkinje cells was the nuclear and cytoplasmic volume deficit the same (−31%). The match between the neuronal and the nuclear volume was not recorded in the version 7.001 of Stereo Investigator that was used. For this reason, the volume of the cytoplasm was calculated as the difference between the volume of the cell body and of the cell nucleus for each structure/subject (Table 2). Therefore, the significance of the difference in the volume of the neuronal cytoplasm could not be determined.

**Table 2:** **The difference between the mean volume of neuronal cytoplasm in autistic and control cohorts**

**Brain structure**	**4- to 8-year-old subjects**		**11- to 23-year-old subjects**		**29- to 64-year-old subjects**	
	**Control**	**Autism**	**Control**	**Autism**	**Control**	**Autism**
	**Mean ± SD (100%)**	**Mean ± SD (%)**	**Mean ± SD (100%)**	**Mean ± SD (%)**	**Mean (SD) (100%)**	**Mean ± SD (%)**
Nucleus accumbens	900 ± 108	615 ± 122 -32%	864 ± 113	808 ± 46 -6%	758 ± 113	849 ± 242 + 12%
Purkinje cells	11,060 ± 466	7,653 ± 2,176 -31%	10,899 ± 1,261	9,222 ± 358 -15%	10,275 ± 1,434	7,735 ± 860 -25%
Claustrum	1,599 ± 167	1,173 ± 230 -27%	1,589 ± 75	1,358 ± 129 -15%	1,518 ± 180	1,415 ± 124 -7%
Thalamus	3,057 ± 220	2,311 ± 225 -24%	2,696 ± 255	2,617 ± 283 -3%	2,768 ± 214	2,954 ± 218 + 7%
Globus pallidus	5,011 ± 190	3,999 ± 336 -20%	4,061 ± 864	3,875 ± 660 -5%	4,523 ± 935	4,441 ± 1,008 -2%
Dentate nucleus	7,734 ± 974	5,913 ± 1,168 -23%	7,883 ± 751	6,617 ± 951 -16%	7,335 ± 516	6,505 ± 2,047 -11%
Entorhinal cortex	1,862 ± 167	1,434 ± 178 -23%	1,948 ± 135	1,597 ± 186 -18%	1,969 ± 102	1,980 ± 432 + 1%
Amygdala	2,427 ± 125	1,878 ± 287 -23%	2,386 ± 56	2,153 ± 282 -10%	2,398 ± 256	2,054 ± 413 -14%
Magnocellular basal complex	7,728 + 475	6,076 ± 302 -21%	7,639 ± 750	7,489 ± 556 -2%	7,867 ± 1,087	7,563 ± 1,028 -4%
Putamen	1,071 ± 105	914 ± 92 -15%	820 ± 163	758 ± 125 -8%	679 ± 98	770 ± 165 + 13%
Caudate nucleus	943 ± 106	754 ± 93 -20%	988 ± 148	958 ± 103 -3%	874 ± 160	983 ± 165 + 12%
Inferior olive	3,852 ± 1,428	3,325 ± 352 -14%	3,136 ± 677	3,619 ± 575 + 15%	3,409 ± 616	3,668 ± 72 + 8%
Magnocellular LGN	4,786 ± 266	4,261 ± 665 -11%	5,010 ± 87	4,855 ± 695 -3%	5,119 ± 655	5,230 ± 473 + 2%
Parvocellular LGN	2,337 ± 280	2,249 ± 316 -4%	2,563 ± 156	2,260 ± 378 -12%	2,547 ± 356	2,614 ± 445 + 3%
Substantia nigra	8,193 ± 1,344	7,847 ± 615 -4%	8,147 ± 487	8,271 ± 730 + 1%	8,679 ± 2,270	10,416 ± 1,146 + 20%
Ammon’s horn	2,712 ± 152	2,444 + 494 -10%	2,972 ± 221	2,515 ± 297 -3%	2,892 ± 452	2,752 ± 718 -5%

Mean cytoplasm volume ± SD; LGN, lateral geniculate nucleus.

### The difference between courses of age-associated changes in nucleus and cytoplasm volume in autistic and control group

**Figure 2:**
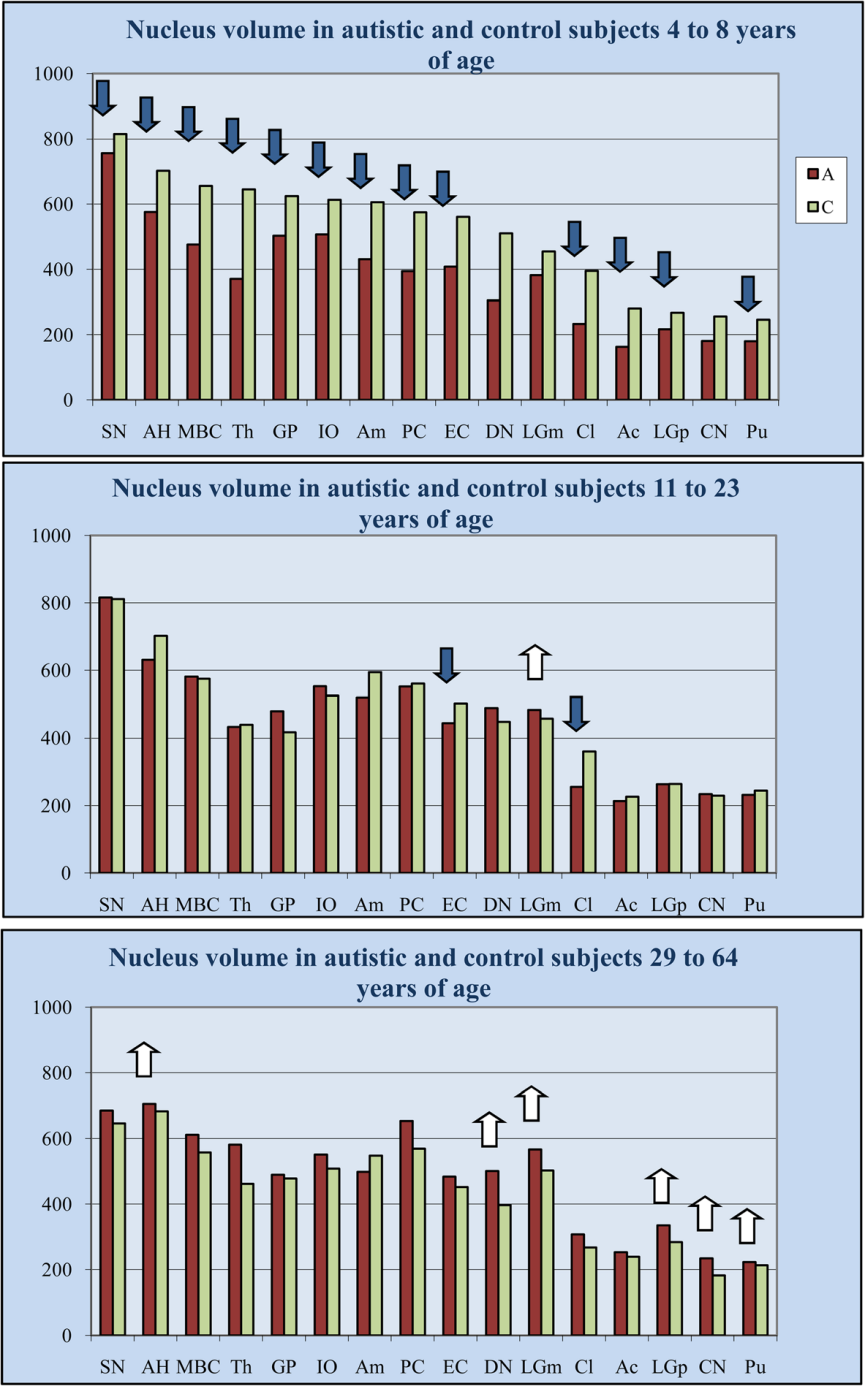
**Alterations of the neuronal nucleus volume deficit throughout the lifespan of subjects diagnosed with autism.** Comparison of neuronal nucleus volume (μm^3^) in 4- to 8-year-old autistic and control subjects revealed significant nucleus volume deficits (blue arrows) in 13 of 16 brain regions examined. In 11- to 23-year-old autistic subjects, the deficit was significant in only two of 16 structures. In 29- to 64-year-old autistic subjects, differences in neuronal nucleus volume were not significant in 10 structures, whereas the volume of nuclei of autistic subjects significantly exceeded the volume of nuclei of control subjects in six other structures (white arrows).

To detect age-associated changes in neuronal nucleus volume throughout the lifespan of autistic subjects, the volume of nuclei in 16 brain regions in 4- to 8-, 11- to 23-, and 29- to 40-year-old autistic subjects was compared with that of age-matched control subjects (Table 1). Whereas in 4- to 8-year-old autistic children, the nuclear volume deficit was significant in 13 of 16 regions, the number of regions with significant deficits was reduced to only two in 11- to 23-year-old autistic individuals (Table 1, Figure 2), and there was no nuclear volume deficit in 29- to 60-year-old autistic subjects. The increase in neuronal nucleus volume with age in autistic subjects is reflected in the nuclei that were larger than control nuclei in the magnocellular layer of the LGN in 11- to 23-year-old autistic subjects, and the significantly larger neuronal nuclei in six of 16 regions in autistic adults 29– 60 years of age, including the Ammon’s horn, dentate nucleus, magnocellular and parvocellular layers of the LGN, caudate nucleus, and putamen. The increase in the seven other regions did not reach significance. These data indicate that the number of regions with a non-significant difference between the neuronal nucleus volume in autistic and control subjects increased from three in children, to 13 in teenagers/young adults, and ten in older adults.

**Figure 3:**
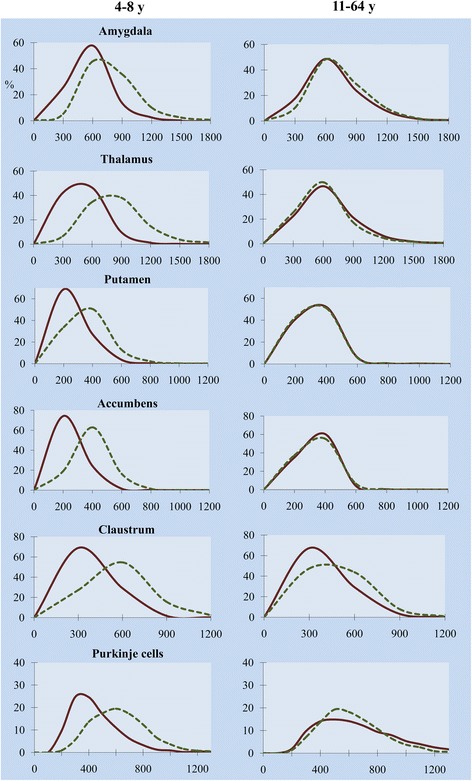
**Multiregional prevalence of small neuronal nuclei in 4- to 8-year-old children with autism.** Frequency distribution of neuronal nucleus volume in six selected structures demonstrates a significant shift towards small neuronal nuclei in 4- to 8-year-old autistic children (continuous line) in comparison to age-matched control subjects (dotted line), but differences between 11- to 64-year-old autistic and control subjects are significantly reduced or are undetectable.

Comparative analysis of the volume of neuronal nucleus frequency distribution in 4- to 8-year-old and 11- to 64-year-old autistic subjects versus age-matched control subjects demonstrates a high percentage of small neuronal nuclei in autistic children. However, in most of the examined regions, the difference between neuronal nucleus volume in adult autistic and in control subjects is significantly reduced or undetectable (Figure 3). These data suggest an increase of neuron nucleus volume in the autistic group and a decrease in the control group.

### Trajectories of neuronal nucleus volume modifications during the lifespan in autistic and control cohorts examined independently

**Figure 4:**
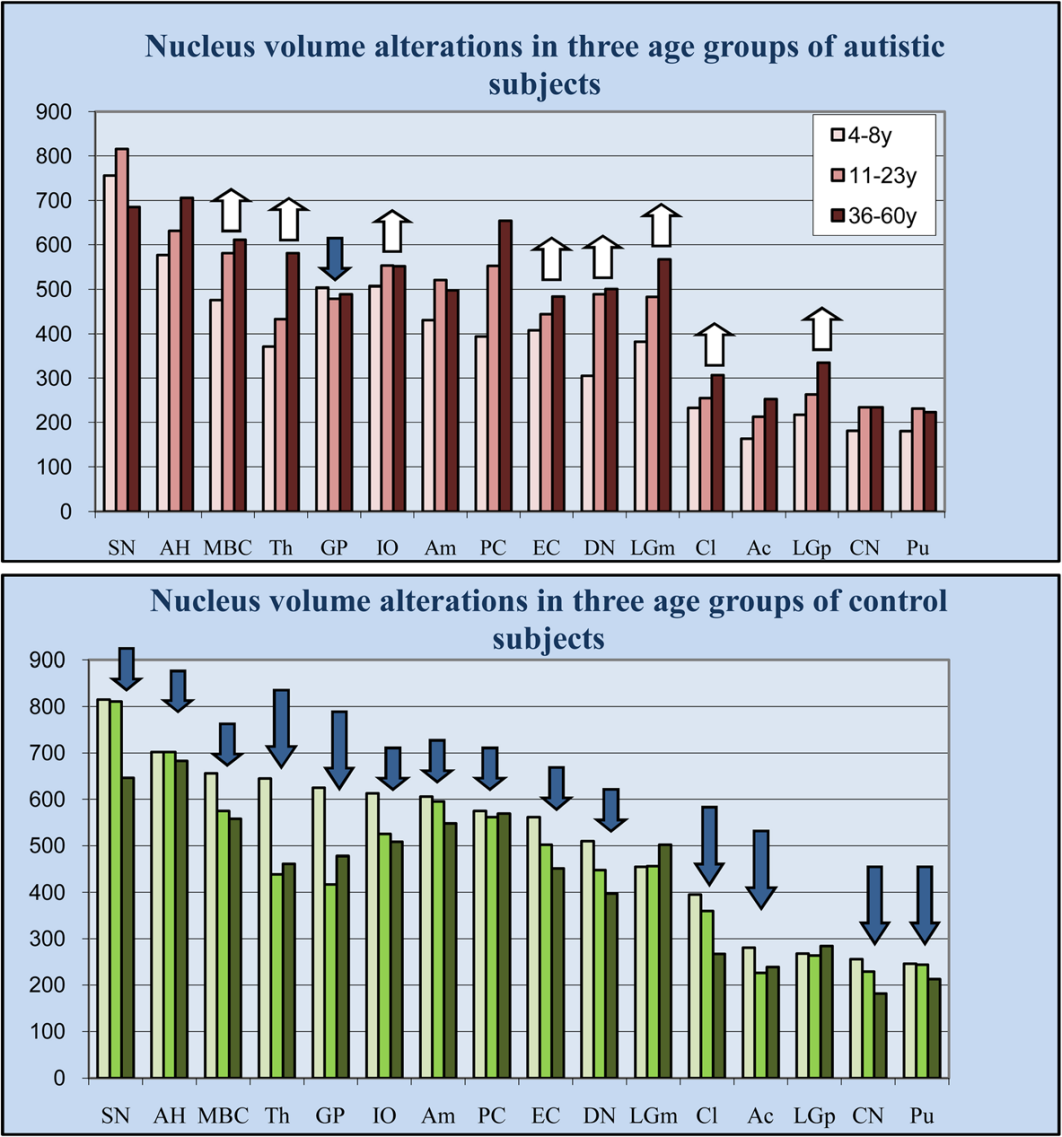
**Increase of neuronal nuclei volume in teenagers and adults with autism, and decrease in control cohort.** Estimates of nucleus volume (μm^3^) in three age groups of subjects with autism revealed significant increase in eight brain regions (white arrows). In the control cohort, the volume of neurons decreased significantly in 14 regions. The significance level was computed by controlling for post-mortem interval, days of fixation, days of dehydration, and brain weight loss during dehydration. Large arrows indicate a significant difference in both age groups; small arrow marks a significant difference in one of two age groups.

To define the differences between the trajectories of age-associated alterations in neuronal nucleus volume in autism and control subjects, the volume of nuclei has been compared in three age groups within the autistic cohort and within the control cohort. The study revealed opposite trends in autistic and control cohorts (Table 3, Figure 4). The dominant feature of the autistic cohort was the significant increase in the neuronal nucleus volume in eight regions in one of the older groups, including the MBC, thalamus, magno- and parvocellular layers of the LGN, inferior olive, entorhinal cortex, dentate nucleus, and claustrum. The increase observed in six other regions (substantia nigra, Ammon’s horn, amygdala, nucleus, accumbens, caudate nucleus, and putamen) and Purkinje cells in cerebellum did not reach significance. A small but significant decrease of neuronal nucleus volume was found only in the globus pallidus.

**Table 3:** **Trajectory of neuronal nucleus volume changes throughout the lifespan of autistic and control subjects**

**Brain region**	**Autism**					**Control**				
	**A**	**B**	**C**	**Diff %** ***P value***	**Diff %** ***P value***	**A**	**B**	**C**	**Diff %** ***P value***	**Diff %** ***P value***
	**4–8 y**	**11–23 y**	**36–60 y**	**A/B**	**A/C**	**4–8 y**	**14–23 y**	**29–64 y**	**A/B**	**A/C**
Nucleus accumbens	163	213	253	31% 0.911	55% 0.904	281	226	239	−20% 0.001	−15% 0.001
Purkinje cells	394	552	654	40% 0.226	66% 0.468	575	561	569	−2% 0.000	−1 0.402
Claustrum	233	255	307	9% 0.081	32% 0.000	395	360	267	−9% 0.001	−32 0.000
Thalamus	371	433	581	17% 0.533	57% 0.000	645	439	462	−32% 0.000	−28% 0.000
Globus pallidus	503	479	489	−5% 0.357	−3% 0.000	625	417	478	−33% 0.001	−24% 0.000
Dentate nucleus	305	489	501	60% 0.257	64% 0.000	510	448	397	−12% 0.269	−22% 0.008
Entorhinal cortex	408	444	484	9% 0.857	19% 0.007	561	502	452	−10% 0.632	−19.6% 0.000
Amygdala	431	520	498	21% 0.140	16% 0.732	606	595	548	−2% 0.001	−10% 0.026
Magnocellular basal complex	476	581	611	22% 0.000	28% 0.030	656	575	558	−12% 0.000	−15% 0.064
Putamen	180	231	223	28% 0.332	24% 0.830	246	244	213	−1% 0.019	−13% 0.007
Caudate nucleus	181	234	234	29% 0.374	29% 0.812	256	229	182	−11% 0.003	−29% 0.000
Inferior olive	507	553	551	9% 0.089	9% 0.000	613	525	508	−14% 0.000	−17% 0.150
Magnocellular LGN	382	483	567	26% 0.405	48% 0.002	455	457	502	0 0.255	10% 0.189
Parvocellular LGN	217	263	335	21% 0.578	54% 0.000	268	264	284	−1% 0.503	6% 0.436
Substantia nigra	756	816	685	8% 0.911	−9% 0.035	815	811	646	0% 0.367	−21% 0.002
Ammon’s horn	576	631	706	9% 0.509	23% 0.888	702	702	683	0% 0.316	−3% 0.000

Significance levels computed controlling for post-mortem interval, days of fixation, days of dehydration, and weight loss. Results were non-significant using a false discovery rate (FDR) of p < 0.05. A/B and A/C not bootstrapped p-value.

The characteristic feature of the control cohort was the decrease in neuronal nucleus volume in one of the two older groups in eight regions and in both older age groups in the six other regions, including the thalamus, globus pallidus, claustrum, nucleus accumbens, caudate nucleus, and putamen. Only two regions (magno- and parvocellular layers of the LGN) did not reveal significant age-associated modifications of the neuronal nucleus volume in control cohorts. In spite of the general shift towards larger nuclei in autistic subjects and towards smaller nuclei in the control group, there are significant inter-regional differences in neuronal volume growth or reduction during the lifespan in autistic and control cohorts.

To explain why very striking differences in the mean volume of neuronal nuclei in the nucleus accumbens of children and teenagers/young adults (31% and 55%, respectively), Purkinje cells (40% and 66%, respectively), dentate nucleus (60%), amygdala (21%), putamen (28% and 24%, respectively), caudate nucleus (29% and 29%, respectively), magnocellular LGN (26%), and parvocellular LGN (21%) are not statistically significant, individual case estimates were analyzed. Comparison of the neuronal nucleus volume in four autistic children 4–8 years of age revealed prominent inter-individual differences in the mean nucleus volume as well as broad variations in the nucleus size within case.

**Figure 5:**
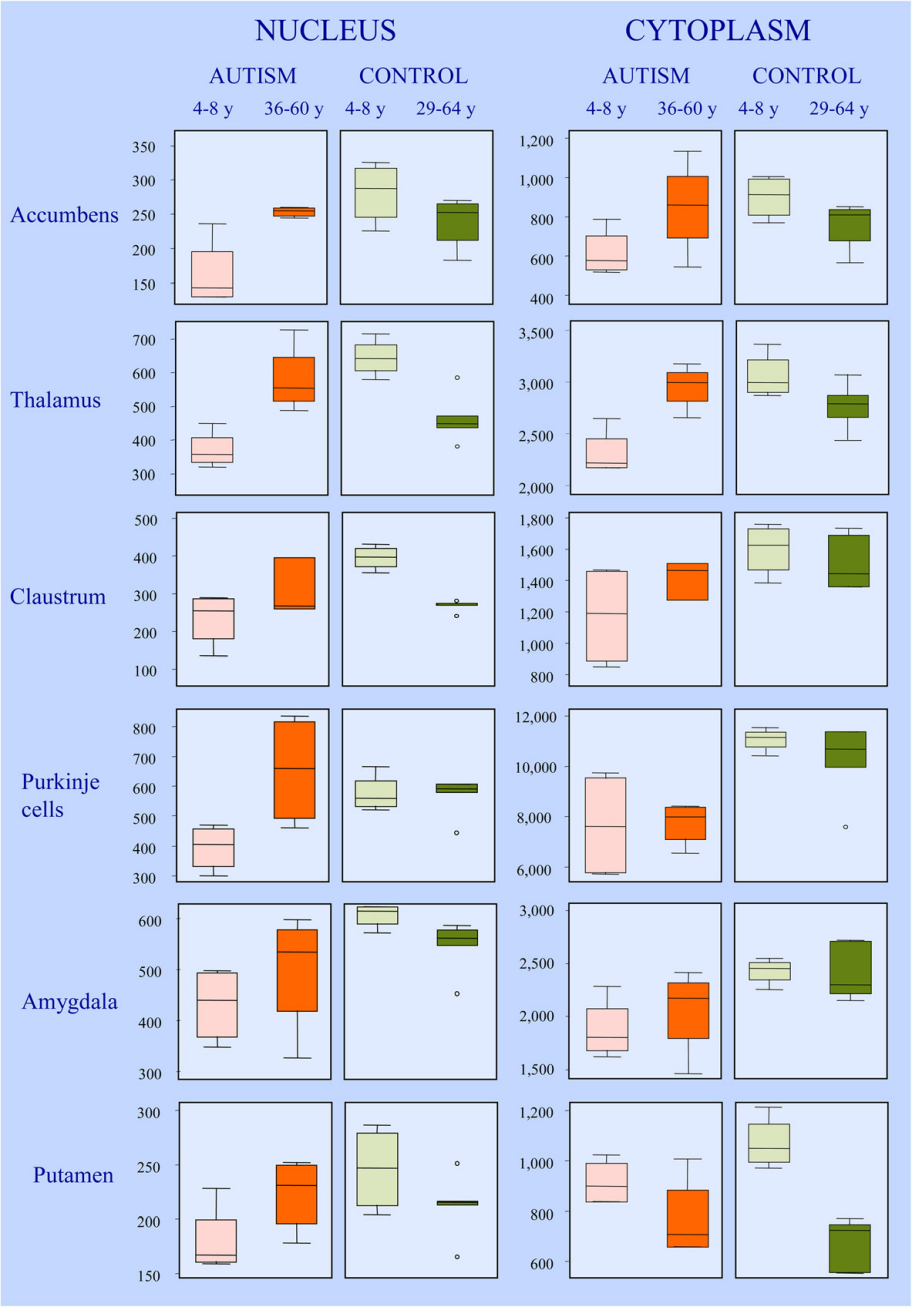
**Difference between neuronal nucleus and cytoplasm volume distribution in control and autism group.** Box plot illustrates neuronal nucleus volume distributions in the six brain structures with the most severe volume deficit. The upper and lower boundaries of each box represent the 75^th^ and 25^th^ percentiles of nucleus volume (μm^3^), respectively. The depth of the box represents the interquartile range (IQR). The whisker above the box marks the maximum value unless any data point lies more than 1.5 times of the IQR above the 75^th^ percentile. The points outside it (outliers) are indicated by circles. The lower whisker and outliers are shown analogously. In all six brain structures, the median volumes of neuronal nuclei in autistic children were below the range of the median volumes of four 4- to 8-year-old control children. The dominant feature in 10 autistic subjects 36–60 years of age was an increase in the volume of nuclei in comparison to in autistic children, as shown as the IQR of older subjects above the median value of children. The level of 75th percentile and the whisker above the box indicate that in the older cohort, the volume of nuclei of the majority of subjects increases with age. The persistence of the volume of neuronal nuclei in the amygdala in a minority of adults at the level of that of 4- to 8-year-old children suggests heterogeneity within the autistic group and indicates a lack of neuronal nucleus increase in some autistic adults. The opposite pattern is observed in the 29- to 64-year-old control group, reflected in reduction of median neuronal nucleus volume and IQR shift below the median value of control 4- to 8-year-old children. In general, the volume of cytoplasm increases with age in adult autistic subjects and decreases in the control group.

The box plot shown in Figure 5 illustrates neuronal nucleus volume distributions in the six brain structures with the most severe volume deficit and demonstrates that in all these structures, the median neuronal nucleus volumes for all four autistic children were below the range of those for the four 4- to 8-year-old control children. In the majority of autistic subjects 36–60 years of age, the neuronal nucleus volume is increased to the range of that in the control group. The box plot demonstrates an opposite pattern of changes in the 29- to 64-year-old control group, with a reduction in the median neuronal nucleus volume in comparison to that in the 4- to 8-year-old control children. The increase in the volume of the neuronal soma cytoplasm in 36- to 60-year-old autistic subjects in comparison to that in autistic children as well as the partial decrease in that volume in adult controls in comparison to in control children replicates the pattern of nuclei alterations in both cohorts.

## Discussion

### Global pattern of abnormal neuron growth in autism

Morphometric studies designed to detect clinicopathological correlations demonstrated links between functional deficits observed in autism, and reduced size of neurons in several cortical regions including superior and middle frontal gyrus [42], inferior frontal cortex (BA 44 and 45) [32], fusiform gyrus [34], anterior midcingulate [43] and anterior cingulate cortex [44], as well as Purkinje cells in the cerebellum [45], and neurons in the brainstem superior olive [46]. In addition, neuropathological studies not supported with morphometric methods revealed small neurons in the amygdala, entorhinal cortex, mammilary body, [47], Ammon’s horn [48], medial septal nucleus [49], cerebellar nuclei and inferior olive [50]. The present study of neuronal nucleus and cytoplasm volumes and our previous study of cell soma volume in 14 brain regions [27], combined with parallel studies of several cortical regions in the same cohort, and neuropathological studies listed in Table 4, disclose the global nature of developmental alterations with multiregional developmental alterations rather than changes limited to a few regions. The detected pattern of developmental alterations of neuronal growth may contribute to the diagnostic features of ASD, as well as a number of associated conditions, including intellectual deficits, depression, obsessive-compulsive disorders, anxiety, oppositional/defiant disorder, aggression, and self-injurious behaviors [51,52]. The absence of significant neuronal soma alterations in cortical regions with little-known relevance to autism, such as posteroinferior occipitotemporal gyrus [53], primary visual cortex [34] and prefrontal cortex [54] support the specificity of neuropathological changes in autism. The broad spectrum of deficits of neuronal soma, nucleus, and cytoplasm volume shown in this study reveals brain region/neuron type-specific sub-patterns of developmental abnormalities in autism.

**Table 4:** **Neuronal soma, nucleus and cytoplasm volume deficit in 4- to 8-year old autistic subjects compared to results of previous qualitative and morphometric studies**

**Brain structure or neuron type**	**Volume deficit in 4- to 8-year-old autistic subjects**			**Alterations of neuron soma size detected in previous studies**
	**Nucleus**	**Cytoplasm**	**Soma**	
**N. accumbens**	−42%*	**−32%**	−34%*	
**Thalamus**	−42%*	**−24%**	−27%*	
**Claustrum**	−41%*	**−27%**	−29%*	
**Purkinje cells**	−31%*	**−31%**	−31%*	24% Purkinje cell area deficit [45]
**Amygdala**	−29%*	**−23%**	−24%*	Reduced neuron soma size in some amygdala nuclei [47]
**Magnocellular basal complex**	−27%*	**−21%**	−22%*	
**Putamen**	−27%*	**−15%**	**−17% ns**	
**Entorhinal cortex**	−27%*	**−23%**	−24%*	Reduced neuron soma size [47]
**Globus pallidus**	−19%*	**−20%**	−20%*	
**Parvocellular LGN**	−19%*	**−4%**	−5%*	
**Ammon’s horn**	−18%*	**−10%**	−12%*	27% neuron volume deficit in CA4 of 9 y old [48]
**Inferior olive**	−17%*	**−14%**	**−14% ns**	Enlarged neurons in less than 12 years old and smaller in over the age of 22 years [50]
**Substantia nigra**	−7%*	**−4%**	−5%*	
**Magnocellular LGN**	**−16% ns**	**−11%**	−12%*	
**Caudate nucleus**	**−29% ns**	**−20%**	−22%*	
**Dentate nucleus**	**−40% ns**	**−23%**	−25%*	Enlarged neurons in less than 12 years old and smaller in over the age of 22 years [50]
**Superior olive**	* Significant neuron volume deficit in medial superior olive [46]			
**Superior and middle frontal gyrus**	*Significantly smaller neurons in six autistic subjects 4 to 24 years of age [42]			
**Inferior frontal cortex (BA 44 and 45)**	*Significant neuron volume deficit by 18% in layer III and V, and by 22% in layer VI [32]			
**Fusiform gyrus**	*Significant neuron volume deficit by 21% in layer V and by 13.4% in layer VI [34]			
**Anterior midcingulate cortex**	*Significant pyramidal neurons volume deficit in young children [43]			
**Posteroinferior occipitotemporal gyrus**	*No significant difference in pyramidal neurons volume [53]			
**Prefrontal cortex**	*Unchanged size of neurons [54]			
Anterior cingulate cortex	Significant neuron volume deficit in layers I-III and V-VI [44]			
Mammillary body	Smaller neurons [47]			
Medial septal nucleus	Smaller neurons [49]			
Fastigial, globose, emboliform nucleus	Enlarged neurons in less than 12 years old and smaller in over the age of 22 years [50]			

The 16 regions marked with bold were examined in this study. The superior olivary complex and six neocortical regions marked with bold (*) were examined by other researchers using significant amount of material from this cohort of 14 autistic and 14 control subjects. The significance of difference of the cytoplasm volume was not determined due to software limitations.

### Patterns of age-associated nucleus and cytoplasm changes throughout the lifespan in autistic and control groups

The present study demonstrates that alterations of neuronal soma volume in autism are associated with alterations of the nucleus and cytoplasm volumes, and that the volumes of these two neuronal body compartments is the lowest in 4- to 8-year-old autistic children. The detected brain region- and neuron type-specific neuronal volume deficit suggests that this developmental abnormality is established before 4 years of age. Because diagnosis is made at the age of 3 years or older, verification of this hypothesis on the basis of current diagnostic criteria is impossible. However, a study of neuronal progenitor cells suggests that in autism, neuronal size might be defined at age much less than 3 years. The exposure of human neuronal progenitor cells originating from the fetal cerebral cortex to media enriched with serum from autistic donors ~3 years of age or from age-matched healthy donors revealed that in colonies that developed in “autistic” conditions, the percentage of smaller cells was significantly higher than in colonies exposed to sera of healthy donors [55].

Whereas nuclear volume deficit was significant in 13 of 16 regions in 4- to 8-year-old autistic children, the number of regions with a significant deficit was reduced to only two in 11- to 23-year-old autistic individuals, and there was no nuclear volume deficit in 29- to 60-year-old autistic subjects. These findings suggest that in autistic subjects, deceleration of neuronal nuclear and cytoplasmic growth during childhood is followed by acceleration of neuronal volume growth during adolescence and early adulthood. The detected pattern explains the differences between characteristics of neuronal size in other reports. In our cohort, the significant difference in volume of neuronal soma (−24%) or nucleus in the amygdala (−29%; p < 0.001) was detected only in the youngest autistic subjects (4–8 years of age), and the difference in nucleus and cell volume was not significant in older groups (11–64 years of age). This result is similar to the result of averaging of the neuronal size in the amygdala of 10- to 44-year-old autistic subjects [56], when the difference is masked by an increase in the neuronal volume in autistic subjects and a decrease in the neuronal volume in control subjects.

### Common mechanisms contributing to smaller neuron size in neurodevelopmental disorders

Autism and Rett syndrome overlap phenotypically and neuropathologically. In both conditions, a similar delayed regression after apparently normal prenatal and early postnatal development is observed [57]. Rett syndrome is caused by over 300 mutations of the *methyl-CpG-binding protein 2 (MECP2)* gene [58,59]. The *MECP2* gene encodes methyl CpG–binding protein-1 (MeCP2), a transcriptional repressor required for proper development of post-migratory neurons, but with cell-specific differences in MeCP2 levels [60]. In Rett syndrome, normal head circumference at birth, but deceleration of growth at 2–3 months of age results in a 12–34% deficit in brain weight and volume [61,62], reduced neuronal size in the cortex, thalamus, basal ganglia, amygdala, and hippocampus [63], decrease in the size of cortical minicolumns [64], and reduced dendritic branching [65]. Furthermore, the reduced size of pyramidal neurons and the lesser complexity of dendritic arborizations in MeCP2 mutant mice indicate that MeCP 2 is involved in maturation and the maintenance of neurons, including dendritic integrity and synaptogenesis [66,67]. The significant reduction in MeCP2 expression in 79% of autism, 100% of Angelman syndrome, 75% of Prader-Willi syndrome, and 60% of Down syndrome cases [57] compared to age-matched controls suggests that altered MeCP2 expression contributes to abnormal postnatal brain development and to an abnormal course of neuron maturation in neurodevelopmental disorders [68].

MeCP2 is an abundant nuclear protein with elevated expression during postnatal brain development [60]. The chromatin-binding function of MeCP2 is required for neuronal nucleus and nucleolus development and maturation. Critical for this binding activity is an intact methyl-binding domain at the amino terminus of the MeCP2 protein [69]. During neuronal maturation, the nuclear morphology changes from a small, heterochromatic nucleus with many randomly located chromocenters and several nucleoli to a large, mostly euchromatic nucleus with fewer and larger chromocenters and a large, centrally located nucleolus [70]. In the absence of MeCP2, the neuronal nuclei fail to increase in size at normal rates during cell differentiation. Neurons lacking MeCP2 have a significantly reduced rate of RNA synthesis; however, re-expressing MeCP2 in mutant neurons rescues the nuclear size phenotype *in vitro* [71] and in *Mecp2* mice [72]. Reduced expression of MeCP2 in the majority of autistic subjects [57] appears to contribute to nucleus volume deficit.

### Alterations that may contribute to neuronal cytoplasm volume changes throughout the lifespan of autistic individuals

One may hypothesize that the increase in neuronal soma, nucleus, and cytoplasm volume in autistic adolescents corresponds to delayed cell development/maturation. However, this assumption appears to be in conflict with biochemistry- and immunocytochemistry-supported neuropathological studies that demonstrate a broad spectrum of pathology in the neuronal energy-generating system, metabolism, degradation and storage systems, and oxidative stress. These alterations affect major cytoplasmic compartments, including mitochondria, endocytic vesicles, lysosomes, and autophagic vacuoles, as well as lipofuscin deposits and suggest that they may directly contribute to the abnormal increase in cytoplasm volume in adolescence/adulthood.

Mitochondria are one of the major compartments of neuronal cytoplasm. Mitochondrial DNA mutations and copy number variations [73-75] support the hypothesis of a mitochondrial pathology in ASD. Developmental alterations include the perturbations of mitochondrial energy generation that have been demonstrated in some individuals diagnosed with ASD [76-80], and mitochondrial respiratory chain dysfunction [75].

Mitochondrial electron transport chain (ETC) complexes generate the proton gradient used by ATP synthase to catalyze ATP formation [81,82]. In autistic subjects, alterations of ETC reveal brain region–specific differences, with a decrease in the ETC protein levels in the cerebellum and frontal and temporal cortices, but not in the occipital and parietal cortices. Chauhan *et al*. [80] reported a significant reduction in respiratory chain protein expression, with lower levels of complexes II, III, and IV in the temporal cortex of autistic children. ETC alterations are associated with age. Children with autism 4–10 years of age have lower levels of ETC proteins than control subjects; however, the difference in subjects from 14–39 years of age was not significant [75,80]. Decreased complexes III and IV were also reported by Tang *et al.* [83].

Cytoplasm volume changes might be directly related to increased mitochondrial mass, as measured by levels of the mitochondrial membrane proteins porin, Tom20, and Tim23, and significant changes in the expression of mitochondrial fission and fusion proteins, suggesting the imbalance between mitochondrial biogenesis and degradation in neurons in the temporal cortex of young ASD subjects [83]. These alterations can change the morphology, number, and function of mitochondria [84]. Autophagosomes degrade damaged mitochondria, producing excessive reactive oxygen species via ubiquitin-mediated recognition and selective targeting [85]. Several ubiquitin genes have been implicated in ASD [86], including PARK2 that encodes parkin, which assists in mitochondria dystruction [87]. However, an increase in compromised mitochondria suggests impaired mitophagy in ASD [83].

Reported mitochondrial alterations reflect the disturbed energy production that coexists with alterations of neuronal metabolism [75,88-90] and the enhanced degradation of DNA, RNA, and proteins by oxidative stress [80,91,92] that may result in enhanced cell organelle turnover [77], excessive accumulation of products of degradation [93], and pathological increase of the cytoplasm and cell volume.

### Enhanced anabolic APP processing in autism

Alternative cleavage of amyloid-β precursor protein (APP) with α secretase releases non-amyloidogenic secreted APPα (sAPPα) [94]. Recent studies revealed enhanced non-amyloidogenic cleavage of APP with α and γ secretases in such developmental disorders as autism and fragile X syndrome (FXS) [88,95-98]. Sokol *et al.* [95] and Ray *et al.* [89] reported two or more times higher levels of total sAPP, including sAPPα, and a decrease in the levels of both Aβ40 and Aβ42 in young children with severe autism in comparison to children without autism. A study of 25 autistic subjects revealed elevated plasma sAPPα in 60% of children with autism (n = 25) compared to healthy controls [98]. The elevated levels of sAPPα in autistic individuals with severe autism but not in neurotypical and mildly autistic subjects suggest a link between levels of sAPPα and autism severity [89].

Neurons in the brain of control children and adults accumulate cell type–specific amounts of Aβ_17–40/42_, which is the product of nonamyloidogenic APP processing with α- and γ-secretase [99]. The enhanced cytoplasmic accumulation of mainly N-terminally truncated Aβ was detected in 11 of 12 examined brain regions in autistic children, adolescents, and adults diagnosed with Dup15q11.2-q.13 (dup15) and in 8 of 12 regions in children, adolescents, and adults with idiopathic autism, including neurons in all three cortical regions, the amygdala, thalamus, Purkinje cells, lateral geniculate nucleus, and dentate nucleus [100]. Enhanced non-amyloidogenic APP metabolism is associated with excessive accumulation of Aβ_17–40/42_ within the organelles involved in proteolysis and storage of metabolic remnants [92-100]. One may hypothesize that the observed neuron type- and region-specific increase in the volume of neuronal cytoplasm is a reflection at least in part of a neuron type- and region-specific increase in sAPPα production and neuron type- and region-specific enhanced accumulation of Aβ_17–40/42_ in several cytoplasmic compartments, including endocytic vesicles, autophagic vacuoles, lysosomes, and lipofuscin, and in minute amounts in mitochondria. Application of Lamp 1 as a lysosomal marker revealed that approximately 20–30% of neuron cytoplasmic Aβ_17–40/42_ is detected in the endolysosomal pathway, whereas another 20–30% of neuronal Aβ_17–40/42_ is accumulated in the lipofuscin [92,100]. An increase in the percentage of Aβ_17–40/42_-accumulating neurons appears to be associated with an increase in the number of lipofuscin-containing neurons observed in our study [100] and in an increase in the number of lipofuscin-containing neurons by 69% in Brodmann area 22, by 149% in area 39, and by 45% in area 44 [93]. The higher prevalence of excessive Aβ_17–40/42_ in autistic subjects with dup(15), the early onset of intractable seizures, and the high risk of SUDEP support reports demonstrating a link between enhanced sAPPα levels and severe autism [88,89,95,98]. The appearance of diffuse plaques in three autistic subjects 39–53 years of age suggests an age-associated risk of alterations of APP processing with enhanced short-form Aβ deposition in the cytoplasm and full length of Aβ in nonfibrillar plaques [101].

Biochemical reports demonstrate an increase in the level of marker of lipid peroxidation (malondialdehyde, MDA) in the plasma of autistic children [102] and in the cerebral cortex and cerebellum [103]. Lipid peroxidation products were detected in all mitochondria and lipofuscin deposits, numerous autophagic vacuoles, and lysosomes. Neuronal Aβ was colocalized with markers of oxidative stress, including 4-hydroxy-2-nonenal (HNA) and MDA, and increased levels of Aβ levels correlated with higher levels of HNE and MDA [92].

It appears that a neuron type- and region-specific pattern of these metabolic changes and oxidative stress contribute to the cytoplasmic organelle damage, degradation and storage of products of degradation in growing lipofuscin deposits, and the cell cytoplasm alterations detected in this study. The prevalence of this pathology in children/young autistic subjects but with no evidence of significant neuronal loss suggests that oxidative damage of cytoplasmic organelles is associated with an increased turnover/repair of affected cell components. In addition, some reports suggest that increased processing of APP may contribute to autistic functional alterations [89,95].

## Conclusions

This first study of the volume of neuronal nucleus and cytoplasm in autistic subjects reveals a brain region– and neuron type–specific deficit in the volume of both cell compartments in affected 4-to 8-year-old children compared to control subjects. The most severe volume deficit in the youngest group suggests that these developmental abnormalities contribute to emergence of the autistic phenotype before the age of three years. Significant pathology in 13 of 16 examined regions reflects the global nature of developmental deficits and their potential contribution to a broad spectrum of clinical autism manifestations. The observed pattern of nuclear and cytoplasmic alterations suggests four neuron type- and brain region-specific phases of changes in neuron morphology: (a) abnormal as well as delayed neuronal nucleus and cytoplasmic growth before the third year of life, (b) severe volume deficit in early childhood (4–8 years) reflecting developmental abnormalities in first three years of life, (c) significant increase in neuronal nucleus and cytoplasmic volume during adolescence, and (d) relatively modest further modifications during adulthood. The increase in nuclear and cytoplasmic volumes close to the control level in the majority of examined regions in teenagers and adults appears to reflect alterations in neuronal energy production, metabolism, and oxidative stress, rather than delayed neuron growth with an adjustment of structure and function.

## Consent

This postmortem study has been performed using anonymized coded brain tissue samples. Selected clinical records were extracted from the anonymized, coded Autism Tissue Program - Autism Speaks database by authorization to the project’s principal investigator.

## References

[1] DiCiccio-Bloom E, Lord C, Zwaigenbaum L, Courchesne E, Dager SR, Schmitz C (2006). The developmental neurobiology of autism spectrum disorder. J Neurosci.

[2] Ozonoff S, Heung K, Byrd R, Hansen R, Hertz-Picciotto I (2008). The onset of autism: Patterns of symptom emergence in the first years of life. Autism Res.

[3] Zwaigenbaum L, Thurm A, Stone W, Baranek G, Bryson S, Iverson J (2007). Studying the emergence of autism spectrum disorders in high-risk infants: methodological and practical issues. J Autism Dev Disord.

[4] Zwaigenbaum L, Bryson S, Rogers T, Roberts W, Brian J, Szatmari P (2005). Behavioral manifestations of autism in the first year of life. Int J Dev Neurosci.

[5] Landa R, Garrett-Mayer E (2006). Development in infants with autism spectrum disorders: a prospective study. J Child Psychol Psychol.

[6] Lord C, Risi S, Lambrecht L, Cook EH, Leventhal BL, DiLavore PC (2000). The autism diagnostic observation schedule-generic: a standard measure of social and communication deficits associated with the spectrum of autism. J Autism Dev Disord.

[7] Smith LE, Maenner MJ, Seltzer MM (2012). Developmental trajectories in adolescents and adults with autism: the case of daily living skills. J Am Acad Child Adolesc Psychiatry.

[8] Courchesne E, Carper R, Akshoomoff N (2003). Evidence of brain overgrowth in the first year of life in autism. JAMA.

[9] Courchesne E, Redcay E, Kennedy DP (2004). The autistic brain: birth through adulthood. Curr Opin Neurol.

[10] Dementieva YA, Vance DD, Donnelly SL, Elston LA, Wolpert CM, Ravan SA (2005). Accelerated head growth in early development of individuals with autism. Pediatr Neurol.

[11] Redcay E, Courchesne E (2005). When is the brain enlarged in autism? A meta-analysis of all brain size reports. Biol Psychiatry.

[12] Kosaka H, Omori M, Munesue T, Ishitobi M, Matsumara Y, Takahashi T (2010). Smaller insula and inferior frontal volumes in young adults with pervasive developmental disorders. Neuroimage.

[13] Stefanatos GA (2008). Regression in autistic spectrum disorders. Neuropsychiatr Rev.

[14] Ozonoff S, Iosif A, Baguio F, Cook IC, Hill MM, Hutman T (2010). A prospective study of the emergence of early behavioral signs of autism. J Am Acad Child Adolesc Psychiatry.

[15] Goldberg WA, Osann K, Filipek PA, Laulhere T, Jarvis K, Modahl C (2003). Language and other regression: assessment and timing. J Autism Dev Disord.

[16] Malhi P, Singhi P (2012). Regression in children with autism spectrum disorders. Indian J Pediatr.

[17] Hansen RL, Ozonoff S, Krakowiak P, Angkustsiri K, Jones C, Deprey LJ (2008). Regression in autism: Prevalence and associated factors in the CHARGE study. Pediatrics.

[18] Dawson G, Munson J, Webb SJ, Nalty T, Abbott R, Toth K (2007). Rate of head growth decelerates and symptoms worsen in the second year of life in autism. Biol Psychiatry.

[19] Wegiel J, Kuchna I, Nowicki K, Imaki H, Wegiel J, Marchi E (2010). The neuropathology of autism: Defects of neurogenesis and neuronal migration, and dysplastic changes. Acta Neuropathol.

[20] Wegiel J, Schanen NC, Cook EH, Sigman M, Brown WT, Kuchna I (2012). Differences between the pattern of developmental abnormalities in autism associated with duplications 15q11.2-q13 and idiopathic autism. J Neuropathol Exp Neurol.

[21] Tuchman R, Rapin I (2002). Epilepsy in autism. Lancet Neurol.

[22] Volkmar FR, Nelson DS (1990). Seizure disorders in autism. J Am Acad Child Adolesc Psychiatry.

[23] Woolfenden S, Sarkozy V, Ridley G, Coory M, Williams K (2012). A systemic review of two outcomes in autism spectrum disorder: epilepsy and mortality. Dev Med Child Neurol.

[24] Tuchman RF, Rapin I (1997). Regression in pervasive developmental disorders: seizures and epileptiform encephalogram correlates. Pediatrics.

[25] Holmes GL (2004). Effects of early seizures on later behavior and epileptogenicity. Ment Retard Dev Disabil Res Rev.

[26] Tuchman R, Hirtz D, Mamounas LA (2013). NINDS epilepsy and autism spectrum disorders workshop report. Neurology.

[27] Wegiel J, Flory M, Kuchna I, Nowicki K, Ma SY, Imaki H (2014). Brain-region-specific alterations of the trajectories of neuronal volume growth throughout the lifespan in autism. Acta Neuropathol Comm.

[28] Edens LJ, White KH, Jevtic P, Li X, Levy DL (2013). Nuclear size regulation: from single cells to development and disease. Trends Cell Biol.

[29] Levy DL, Heald R (2012). Mechanisms of intracellular scaling. Annu Rev Cell Dev Biol.

[30] Centers for Disease Control and Prevention. Prevalence of Autism Spectrum Disorder Among Children Aged 8 years. Autism and Developmental Disabilities Monitoring Network, 11 sites, United States, 2010. MMWR 2014, 63:1–21. 24670961

[31] Casanova MF, El-Baz AS, Kamat SS, Dombroski BA, Khalifa F, Elnakib A (2013). Focal cortical dysplasias in autism spectrum disorders. Acta Neuropathol Comm.

[32] Jacot-Descombes S, Uppal N, Wicinski B, Santos M, Schmeidler J, Giannakopoulos P (2012). Decreased pyramidal neuron size in Brodmann areas 44 and 45 in patients with autism. Acta Neuropathol.

[33] Santos M, Uppal N, Butti C, Wicinski B, Schmeidler J, Giannakopoulos P (2011). Von economo neurons in autism: a stereologic study of the frontoinsular cortex in children. Brain Res.

[34] van Kooten IAJ, Palmen SJMC, von Cappeln P, Steinbusch HWM, Korr H, Heinsen H (2008). Neurons in the fusiform gyrus are fewer and smaller in autism. Brain.

[35] Wegiel J, Flory M, Kuchna I, Nowicki K, Ma SY, Imaki H (2014). Stereological study of the neuronal number and volume of 38 brain subdivisions of autistic subjects reveals significant alterations restricted to the striatum, amygdale and cerebellum. Acta Neuropathol Comm.

[36] Heinsen H, Arzberger T, Schmitz C (2000). Celloidin mounting (embedding without infiltration)—a new, simple and reliable method for producing serial sections of high thickness through complete human brains and its application to stereological and immunohistochemical investigations. J Chem Neuroanat.

[37] Gundersen HJ (1988). The nucleator. J Microsc.

[38] Gundersen HJG, Jensen EBV (1999). The efficiency of systematic sampling —reconsidered. J Micr.

[39] Benjamini Y, Hochberg Y (1995). Controlling the false discovery rate: a practical and powerful approach to multiple testing. J Royal Stat Soc N57.

[40] StataCorp (2009). Stata: Release 11. Statistical Software.

[41] StataCorp (2011). Stata: Release 12. Statistical Software.

[42] Casanova MF, van Kooten IAJ, Switala AE, van Engeland H, Heinsen H, Steinbusch HW (2006). Minicolumnar abnormalities in autism. Acta Neuropathol.

[43] Uppal N, Wicinski B, Buxbaum JD, Heinsen H, Schmitz C, Hof PR (2014). Neuropathology of the anterior midcingulate cortex in young children with autism. J Neuropathol Exp Neurol.

[44] Simms ML, Kemper TL, Timbie CM, Bauman ML, Blatt GJ (2009). The anterior cingulate cortex in autism: Heterogeneity of qualitative and quantitative cytoarchitectonic features suggests possible subgroups. Acta Neuropathol.

[45] Fatemi SH, Halt AR, Realmuto G, Earle J, Kist DA, Thuras P (2002). Purkinje cell size is reduced in cerebellum of patients with autism. Cell Mol Neurobiol.

[46] Kulesza RJ, Lukose R, Stevens LV (2011). Malformation of the human superior olive in autistic spectrum disorders. Brain Res.

[47] Bauman ML, Kemper TL (1985). Histoanatomic observations of the brain in early infantile autism. Neurology.

[48] Raymond G, Bauman ML, Kemper TL (1996). The hippocampus in autism: Golgi analysis. Ann Neurol.

[49] Kemper TL, Bauman M (1998). Neuropathology of infantile autism. J Neuropath Exp Neurol.

[50] Bauman ML, Kemper TL, Bauman ML, Kemper TL (1994). Neuroanatomic observations of the brain in autism. The Neurobiology of Autism.

[51] Baghdadli A, Pascal C, Grisi S, Aussilloux C (2003). Risk factors for self-injurious behaviours among 222 young children with autistic disorders. J Intell Disabil Res.

[52] Simonoff E, Pickles A, Charman T, Chandler S, Loucas T, Baird G (2008). Psychiatric disorders in children with autism spectrum disorders: prevalence, comorbidity, and associated factors in a population-derived sample. J Am Acad Child Adolesc Psychiatry.

[53] Uppal N, Gianatiempo I, Wicinski B, Schmeidler J, Heinsen H, Schmitz C (2014). Neuropathology of the posterior occipitotemporal gyrus in children with autism. Molec Autism.

[54] Courchesne E, Mouton PR, Calhoun ME, Semendeferi K, Ahrens-Barbeau C, Hallet MJ (2011). Neuron number and size in prefrontal cortex of children with autism. JAMA.

[55] Mazur-Kolecka B, Cohen IL, Jenkins EC, Kaczmarski W, Flory M, Frackowiak J (2007). Altered development of neuronal progenitor cells after stimulation with autistic blood. Brain Res.

[56] Schumann CM, Amaral DG (2006). Stereological analysis of amygdala neuron number in autism. J Neurosci.

[57] Nagarajan RP, Hogart AR, Gwye Y, Martin MR, LaSalle JM (2006). Reduced MeCP2 expression is frequent in autism frontal cortex and correlates with aberrant MECP2 promoter methylation. Epigenetics.

[58] Amir RE, Van den Veyver IB, Wan M, Tran CQ, Francke U, Zoghbi HY (1999). Rett syndrome is caused by mutations in X-linked MECP2, encoding methyl-CpG-binding protein 2. Nat Genet.

[59] Christodoulou J, Grimm A, Maher T, Bennetts B (2003). RettBASE: the IRSA MECP2 variation database—a new mutation database in evolution. Hum Mutat.

[60] Shahbazian MD, Antalaffy B, Armstrong DL, Zoghbi HY (2002). Insight into Rett syndrome: MeCP2 levels display tissue- and cell-specific differences and correlate with neuronal maturation. Hum Mol Genet.

[61] Jellinger K, Armstrong D, Zoghbi HY, Percy AK (1988). Neuropathology of Rett syndrome. Acta Neuropathol.

[62] Reiss AL, Faruque F, Naudy S, Abrams M, Beaty T, Bryan RN (1993). Neuroanatomy of Rett syndrome: A volumetric imaging study. Ann Neurol.

[63] Kaufmann WE, Moser HW (2000). Dendritic anomalies in disorders associated with mental retardation. Cereb Cortex.

[64] Casanova MF, Buxhoeveden D, Switala A, Roy E (2003). Rett syndrome as a minicolumnopathy. Clin Neuropathol.

[65] Armstrong DD (2005). Neuropathology of Rett syndrome. J Child Neurol.

[66] Kishi N, Macklis JD (2004). MECP2 is progressively expressed in post-migratory neurons and is involved in neuronal maturation rather than cell fate decisions. Mol Cell Neurosci.

[67] Fukuda T, Itoh M, Ichikawa T, Washiyama K, Goto Y (2005). Delayed maturation of neuronal architecture and synaptogenesis in cerebral cortex of *Mecp2*-deficient mice. J Neuropathol Exp Neurol.

[68] Gonzales ML, LaSalle JM (2010). The role of MeCP2 in brain development and neurodevelopmental disorders. Curr Psychiatry Rep.

[69] Nan X, Meehan RR, Bird A (1993). Dissection of the methyl-CpG binding domain from the chromosomal protein MeCP2. Nucleic Acids Res.

[70] Singleton MK, Gonzales ML, Leung KN, Yasui DH, Schroeder DI, Dunaway K (2011). MeCP2 is required for global heterochromatic and nucleolar changes during activity-dependent neuronal maturation. Neurobiol Dis.

[71] Yazdani M, Deogracias R, Guy J, Poot RA, Bird A, Barde YA (2012). Disease modeling using embryonic stem cells: MeCP2 regulates nuclear size and RNA synthesis in neurons. Stem Cells.

[72] Giacometti E, Luikenhuis S, Beard C, Jaenish R (2007). Partial rescue of MeCP2 deficiency by postnatal activation of MeCP2. Proc Natl Acad Sci U S A.

[73] Fillano JJ, Goldenthal MJ, Rhodes CH, Marin-Garcia J (2002). Mitochondrial dysfunction in patients with hypotonia, epilepsy, autism, and developmental delay: HEADD syndrome. J Child Neurol.

[74] Graf WD, Marin-Garcia J, Gao HG, Pizzo S, Naviaux RK, Markusic D (2000). Autism associated with the mitochondrial DNA G8363A transfer RNA(Lys) mutation. J Child Neurol.

[75] Gu F, Chauhan V, Kaur K, Brown WT, LaFauci G, Wegiel J (2013). Alterations in mitochondrial DNA copy number and the activities of electron transport chain complexes and pyruvate dehydrogenase in the frontal cortex from subjects with autism. Transl Psychiatry.

[76] Chugani DC, Sundram BS, Behen M, Lee ML, Moore GJ (1999). Evidence of altered energy metabolism in autistic children. Prog Neuropsychopharmacol Biol Psychiatry.

[77] Filipek PA, Juranek J, Smith M, Mays LZ, Ramos ER, Bocian M (2003). Mitochondrial dysfunction in autistic patients with 15q inverted duplication. Ann Neurol.

[78] Oliveira G, Diogo L, Grazina M, Garcia P, Ataide A, Marques C (2005). Mitochondrial dysfunction in autism spectrum disorders: a population-based study. Dev Med Child Neurol.

[79] Giulivi C, Zhang Y-F, Omanska-Klusek A, Ross-Inta C, Wong S, Hertz-Picciotto I (2010). Mitochondrial dysfunction in autism. JAMA.

[80] Chauhan A, Gu F, Essa MM, Wegiel J, Kaur K, Brown WT (2011). Brain region-specific deficit in mitochondrial electron transport chain complex in children with autism. J Neurochem.

[81] Scholes TA, Hinkle PC (1984). Energetics of ATP-driven reverse electron transfer from cytochrome c to fumarate and from succinate to NAD in submitochondrial particles. Biochemistry.

[82] Bertram R, Gram PM, Luciani DS, Sherman A (2006). A simplified model for mitochondrial ATP production. J Theor Biol.

[83] Tang G, Rios PG, Kuo S-H, Akman HO, Rosoklija G, Tanji K (2013). Mitochondrial abnormalities in temporal lobe of autistic brain. Neurobiol Dis.

[84] Han X-J, Tomizawa K, Fujimura A, Ohmori I, Nishiki T, Matsushita M (2011). Regulation of mitochondrial dynamics and neurodegenerative diseases. Acta Med Okayama.

[85] Kraft C, Peter M, Hofmann K (2010). Selective autophagy: Ubiquitin-mediated recognition and beyond. Nat Cell Biol.

[86] Glessner JT, Wang K, Cai G, Korvatska O, Kim CE, Wood S (2009). Autism genome-wide copy number variation reveals ubiquitin and neuronal genes. Nature.

[87] Scheuerle A, Wilson K (2011). PARK2 copy number aberrations in two children presenting with autism spectrum disorder: further support of an association and possible evidence for a new microdeletion/microduplication syndrome. Am J Med Genet B Neuropsychiatr Genet.

[88] Sokol DK, Maloney B, Long JM, Ray B, Lahiri DK (2011). Autism, Alzheimer disease, and fragile X. APP, FMRP, and mGluR5 are molecular links. Neurology.

[89] Ray B, Long JM, Sokol DK, Lahiri DK (2011). Increased secreted amyloid precursor protein-α (sAPPα) in severe autism: proposal of a specific, anabolic pathway and putative biomarker. PLoS One.

[90] Westmark CJ (2013). What’s happening at synapses? The role of amyloid β-protein precursor and β-amyloid in neurological disorders. Mol Psychiatry.

[91] Sajdel-Sulkowska EM, Xu M, Koibuchi N (2009). Increase in cerebellar neurotrophin-3 and oxidative stress markers in autism. Cerebellum.

[92] Frackowiak J, Mazur-Kolecka B, Schanen NC, Brown WT, Wegiel J (2013). The link between intraneuronal N-truncated amyloid b-peptide and oxidatively modified lipids in idiopathic autism and dup(15q11.2-q13)/autism. Acta Neuropathol Comm.

[93] Lopez-Hurtado E, Prieto JJ (2008). A microscopic study of language-related cortex in autism. Am J Biochem Biotech.

[94] Hardy J (2009). The amyloid hypothesis of Alzheimer’s disease: a critical reappraisal. J Neurochem.

[95] Sokol DK, Chen D, Farlow MR, Dunn DW, Maloney B, Zimmer JA (2006). High levels of Alzheimer-beta amyloid precursor protein (APP) in children with severely autistic behavior and aggression. J Child Neurol.

[96] Westmark CJ, Malter JS (2007). FMRP mediates mGluR5-dependent translation of amyloid precursor protein. PLoS One.

[97] Westmark CJ, Westmark PR, O’Riordan KJ, Ray BC, Hervey CM, Salamat MS (2011). Reversal of fragile X phenotypes by manipulation of AβPP/Aβ levels in *Fmr1*^*KO*^ mice. PLoS One.

[98] Bailey AR, Giunta BN, Obregon D, Nikolic WV, Tian J, Sanberg CD (2008). Peripheral biomarkers in autism: secreted amyloid precursor protein-α as a probable key player in early diagnosis. Int J Clin Exp Med.

[99] Wegiel J, Kuchna I, Nowicki K, Frackowiak J, Mazur-Kolecka B, Imaki H (2007). Intraneuronal Aβ immunoreactivity is not a predictor of brain amyloidosis-β or neurofibrillary degeneration. Acta Neuropathol.

[100] Wegiel J, Frackowiak J, Mazur-Kolecka B, Schanen NC, Cook EH, Sigman M (2012). Abnormal intracellular accumulation and extracellular Aβ deposition in idiopathic and Dup15q11.2-q13 autism spectrum disorders. PLoS One.

[101] Wegiel J, Kuchna I, Nowicki K, Imaki H, Wegiel J, Ma SY (2013). Contribution of olivo-floccular circuitry developmental defects to atypical gaze in autism. Brain Res.

[102] Chauhan A, Chauhan V, Brown WT, Cohen I (2004). Oxidative stress in autism: increased lipid peroxidation and reduced serum levels of ceruloplasmin and transferring—the antioxidant proteins. Life Sci.

[103] Chauhan V, Chauhan A, Chauhan A, Chauhan V, Brown WT (2010). Abnormalities in membrane lipids, membrane-associated proteins, and signal transduction in autism. Autism. Oxidative Stress, Inflammation and Immune Abnormalities.

